# Identifying Key Predictors of Nursing Workload in Emergency Infusion Rooms: A Decision Tree Approach

**DOI:** 10.3390/healthcare14131966

**Published:** 2026-07-02

**Authors:** Leiming Gao, Ruixin Shi, Liuzi Wang, Shengzhi Jiao, Bei Wang

**Affiliations:** School of Nursing, Nanjing University of Chinese Medicine, Nanjing 210023, China

**Keywords:** nursing workload, decision tree approach, emergency department

## Abstract

**Purpose:** Accurate assessment of nursing workload is essential for staffing allocation and operational management in emergency infusion rooms. However, workload generation is influenced by complex and potentially nonlinear interactions among patient volume, treatment duration, and care activities, which may not be adequately captured by conventional statistical approaches. This study aimed to identify key predictors associated with nursing workload intensity and develop an interpretable workload stratification framework using a Classification and Regression Tree (CRT) model. **Methods:** Daily operational data were collected from an emergency infusion room between July 2023 and August 2025. Daily chair utilization rate was used as a proxy indicator of workload intensity. Candidate predictors included total infusion duration, direct care encounters, number of patients receiving infusions, medication dispensing time, severe dependency, fall-risk patients, and triage-level patient volume. A CRT model was developed to identify hierarchical predictor relationships and threshold-based workload classification rules. Model robustness was evaluated using 10-fold cross-validation, comparative analyses with multiple linear regression, random forest, and gradient boosting models, and sensitivity analyses excluding total infusion duration. **Results:** The analysis included 761 valid observation days. Total infusion duration emerged as the most influential predictor, followed by direct care encounters and the number of patients receiving infusions. The CRT model identified clinically interpretable workload thresholds and generated a parsimonious decision structure for workload stratification. Re-substitution and cross-validation risk estimates were 0.045 (SE = 0.005) and 0.046 (SE = 0.005), respectively, indicating stable model performance. Although random forest and gradient boosting achieved higher predictive accuracy, the CRT model provided greater interpretability through transparent decision rules. Sensitivity analyses demonstrated that the overall workload stratification pattern remained largely unchanged after excluding total infusion duration. **Conclusions:** The CRT model identified total infusion duration, direct care encounters, and patient volume as key predictors associated with workload intensity in emergency infusion rooms. Although alternative models achieved higher predictive performance, the CRT approach provided interpretable workload stratification rules that may support staffing allocation and operational decision-making. The findings offer a practical data-driven framework for workload assessment in infusion care settings.

## 1. Introduction

Nursing workload comprises the time, physical effort, and cognitive demand required for nursing activities, including technical complexity and associated risks [1]. Nursing workload is a multidimensional construct encompassing direct patient care, indirect nursing activities, documentation responsibilities, and organizational tasks, all of which contribute to overall nursing demand and resource utilization [2,3,4]. The infusion room is a critical outpatient service unit, which presents unique challenges due to its distinct workflow compared with inpatient wards, limiting the applicability of conventional ward-based workload evaluations.

Existing nursing workload evaluation approaches, including task-based measurement systems and technology-assisted monitoring tools, may still inadequately capture the complexity and dynamic variability of clinical nursing demands [5]. Recent studies have increasingly emphasized the integration of nursing time, care complexity, and technology-assisted workload assessment to improve the accuracy and responsiveness of workload evaluation systems [1,6]. However, these approaches lack adaptability to dynamic clinical environments, such as infusion rooms. The emergency infusion room is a high-intensity clinical setting where nursing workload is influenced by a complex interplay of factors including patient criticality, infusion type, patient turnover rate, and indirect nursing activities. Accurate, real-time assessment of this workload is crucial for effectively allocating nursing staff, reducing occupational burnout, and maintaining nursing quality.

Although nursing workload is a multidimensional construct, direct measurement of overall workload remains challenging in dynamic infusion settings. Therefore, daily chair utilization rate was adopted as a proxy indicator of workload intensity in the present study. Chair utilization rate is widely used as an operational metric for evaluating infusion center capacity and productivity because it reflects the proportion of occupied chair time relative to available chair capacity. Previous studies [7,8] have shown that chair utilization is closely associated with patient flow, treatment duration, staffing demand, and workflow efficiency in infusion services. Accordingly, although chair utilization does not directly measure nursing workload, it provides a practical and routinely available indicator of workload intensity and operational demand in emergency infusion rooms.

Previous studies [9,10] examining nursing workload have primarily relied on conventional statistical approaches, such as correlation analysis and linear regression. However, workload generation is often influenced by nonlinear relationships, complex interactions among operational factors, and hierarchical decision patterns that may not be adequately represented by traditional linear models. Recent studies [11,12,13] have suggested that machine-learning approaches may better capture the multidimensional and dynamic characteristics of healthcare workload and operational demand, particularly in complex clinical environments characterized by heterogeneous patient populations and fluctuating service requirements. Decision tree algorithms offer a promising alternative due to their ability to model nonlinear relationships, process multidimensional data, and generate intuitive classification rules directly applicable to clinical settings [14]. Recent studies have increasingly explored machine learning approaches for healthcare workload prediction and emergency resource optimization. For example, Song et al. applied machine learning models to predict nursing workload in digestive wards and demonstrated improved workload estimation performance [15]. Similarly, El Ariss et al. developed a machine-learning-based framework for emergency department resource allocation, highlighting the value of predictive analytics for staffing optimization [16]. Recent advances in explainable artificial intelligence have further emphasized the importance of interpretable prediction models in emergency care environments [17,18]. Interpretable machine-learning approaches allow clinicians and managers to understand the rationale underlying model predictions, thereby facilitating trust, accountability, and practical implementation. In nursing management and healthcare operations, explainable models are particularly important because workforce allocation decisions often require clear justification and stakeholder acceptance [19,20,21]. However, despite these developments, evidence regarding interpretable machine learning approaches specifically tailored to emergency infusion room nursing workload evaluation remains limited. The predictive accuracy and hierarchical importance of key infusion-specific factors—such as infusion duration, patient dependency level, and triage level—remain to be explored. Decision trees have high efficiency in extracting key predictors and generating interpretable classification rules, and thus they are widely used in many fields, ranging from business modeling to medical diagnostics [22]. Their stratified structure prioritizes variables by predictive importance [23], providing a transparent framework for workload analysis.

Despite increasing interest in nursing workload management, limited evidence has explored the application of interpretable machine-learning approaches to workload assessment in emergency infusion rooms. Furthermore, few studies have established operationally applicable models capable of identifying key predictors and threshold-based workload patterns in this setting. Therefore, this study aimed to develop and validate a classification and regression tree (CRT) model to identify key predictors associated with nursing workload intensity, represented by daily chair utilization rate, in an emergency infusion room. In addition, we sought to establish interpretable workload stratification rules that may support staffing allocation and operational decision-making in clinical practice.

## 2. Literature Review

### 2.1. Nursing Workload in Emergency Settings

Nursing workload is a multidimensional construct involving direct patient care, indirect nursing activities, documentation, communication, technical procedures, and organizational tasks. Existing studies have developed and validated several nursing workload measurement tools, including intervention-based workload scales and multicenter workload assessment instruments, which provide important methodological foundations for quantifying nursing demand in clinical settings.

Previous studies examining nursing workload have commonly relied on conventional statistical approaches, including correlation analysis, linear regression, and multivariate regression models, to explore associations between patient characteristics, staffing factors, workflow variables, and perceived workload. For example, Ivziku et al. [10] identified patient acuity, staffing resources, patient transfers, documentation, isolation, and unscheduled activities as predictors of perceived nursing workload using regression models. Similarly, Griffiths et al. [24] demonstrated that nursing workload and staffing variation were significantly associated with patient outcomes, highlighting the complexity of workload generation and its implications for healthcare quality. These studies provide valuable evidence for understanding workload-related factors; however, they may not fully capture nonlinear relationships and interaction effects among multiple operational indicators.

In infusion-related settings, several studies have attempted to quantify nursing workload using technology-assisted or task-based approaches. For instance, Huiqing [25] used Personal Digital Assistants (PDAs) to quantify infusion-specific tasks (e.g., drug dispensing and venipuncture), and linked these metrics to performance appraisal, demonstrating enhanced nurse motivation and management quality. Juanjuan [26] identified daily patient volume and peak workload as critical indicators, showing that quantitative models reduce errors and increase stakeholder satisfaction. Based on this study, Junyu [27] used a Mobile Nursing Information System (MNIS) to measure the clinical workload (e.g., IV insertion and resuscitation), underscoring the value of informatics in achieving accurate nurse-centric workload evaluations. However, both approaches share a common limitation: they primarily focus on direct, observable tasks (such as venipuncture and resuscitation), potentially overlooking indirect nursing activities (such as paperwork, family communication, and equipment handling) that constitute a significant portion of the overall nursing workload.

These studies reveal a shift toward data-driven workload metrics in infusion care. However, a lack of standardization persists, and existing methods often overlook the hierarchical relationships between workload predictors. The aforementioned tools share a common limitation: they struggle to capture the inherent complexity of infusion room workload. Three characteristics render traditional approaches inadequate:

Nonlinearity. Workload spikes triggered by external events—such as mass casualties—defy accurate prediction by linear models.

Interactive effects. Simultaneously managing critically dependent patients while preparing multiple high-risk infusions creates compound demands far exceeding simple addition.

Hierarchical heterogeneity. Patient needs vary dramatically within the same triage level—a Level 3 patient requiring a single antibiotic infusion versus multiple concurrent infusions presents vastly different workload demands.

These characteristics—nonlinearity, interaction effects, and heterogeneity—are consistent with the analytical strengths of decision tree methods. Decision trees recursively partition data based on significant predictors, capturing nonlinear relationships without requiring predefined functional forms. Their hierarchical structure mirrors patient severity stratification, while intuitive if-then rules provide the interpretability needed for clinical management decisions.

We further clarified that information systems and mobile nursing platforms were not discussed as technological innovations alone, but as tools for capturing workload-related data, quantifying nursing activities, and supporting objective workload assessment and performance evaluation. Recent advances in digital health technologies and machine learning-based workload monitoring systems have further expanded the possibilities for real-time nursing workload assessment and dynamic workforce management in high-intensity clinical settings [5,6].

### 2.2. Decision Tree Applications in Healthcare and Operational Management

Recent studies have increasingly applied machine-learning approaches to healthcare workload prediction, patient flow management, and resource allocation. Song et al. [15] used machine-learning algorithms to dynamically predict nursing workload in digestive wards based on patient characteristics, demonstrating the feasibility of machine-learning methods for nursing workload prediction. Moreno-Sánchez et al. [17] applied explainable artificial intelligence to predict patient flow in emergency departments, supporting the value of interpretable models in emergency resource allocation. Mafirakureva [28] modeled the cost-effectiveness of pediatric tuberculosis (TB) service integration in Africa, using decision trees to analyze outcomes, such as the Disability-Adjusted Life Year (DALY) and mortality; the authors reported that results depend on baseline service coverage. Tesfa [29] used this method to predict pregnancy termination factors in East Africa, reaching a model accuracy of 71.8%, which identified socioeconomic variables (e.g., education and marital status) as key influencers. Juntao [30] demonstrated the effectiveness of decision trees in surgical wards, where admission rates emerged as the primary workload predictor. These studies suggest that machine-learning methods may provide flexible tools for modeling complex healthcare processes involving multidimensional and dynamic clinical data.

Among machine-learning methods, decision tree algorithms are particularly useful when interpretability and operational applicability are important. Unlike black-box models, decision trees generate transparent threshold-based rules that can be directly understood by clinicians and nursing managers. This feature is especially valuable in nursing management, where staffing decisions require clear and explainable criteria. Comparative studies have shown that ensemble-learning methods such as Random Forest and Gradient Boosting often achieve superior predictive performance compared with single decision trees because they reduce variance and improve generalization [31]. However, these improvements are typically achieved at the expense of model transparency and interpretability. In healthcare settings, where model output may influence staffing decisions and operational management [32], interpretable models remain valuable because their decision pathways can be readily understood and validated by clinical stakeholders.

Decision tree models have been applied in various healthcare contexts, including clinical prediction, patient classification stratification, emergency department triage, and resource planning. However, limited evidence has explored the application of decision tree regression models to nursing workload stratification in emergency infusion rooms. More specifically, few studies have investigated whether routinely collected infusion-room indicators can be used to identify interpretable threshold rules for workload intensity. Therefore, applying a classification and regression tree model in this setting may help identify key predictors, clarify their hierarchical contributions, and provide operationally meaningful rules for nursing workload management.

Prior to initiating the study, a systematic literature search was conducted to ascertain the novelty of applying decision tree regression models to the emergency infusion room context. The search was performed across PubMed, CINAHL, Web of Science and the China National Knowledge Infrastructure (CNKI) databases, covering all records from inception through June 2025. The search strategy employed combinations of the following keywords: “decision tree,” “regression tree,” “CART,” “nursing workload,” “emergency department,” “infusion room,” and “outpatient infusion.” The search results confirmed that while machine learning methods, including decision trees, have been increasingly applied to predict patient volumes and acuity in general emergency departments, limited peer-reviewed evidence was identified regarding the use of decision tree regression algorithms to model or predict nursing workload indicators within emergency infusion rooms. More precisely, this paper leverages the effectiveness of decision trees to identify dominant workload indicators (e.g., infusion duration and patient volume) and establish a transparent rule-based framework for resource allocation, aligning with value-based healthcare principles.

## 3. Methodology

### 3.1. Data Collection

This study retrospectively analyzed daily workload data from the infusion room of a Grade-A tertiary hospital in Jiangsu Province, China, covering the period from 1 July 2023 to 31 August 2025. The workload indicators comprise clinical activity metrics (i.e., the daily chair utilization rate, direct care encounters, number of patients receiving infusions, patient transfers, resuscitations, and deaths), patient complexity metrics (counts of high fall-risk patients, patient volume by triage levels, including Levels II, III, and IV, and patients with severe dependency), and time-based metrics (total daily infusion duration and medication dispensing time).

Daily chair utilization rate was calculated as: (total chair occupancy time/total available chair time) × 100%.

The data are provided by Hospital Information System (HIS) (automatically recorded metrics such as infused patients, direct care episodes, and dispensing time) and nurse manager reports (manually documented high-risk and high-dependency cases).

From an initial 792-day dataset, 31 days are excluded due to missing or incomplete records, yielding 761 valid days for analysis. Missing data were assessed using Little’s MCAR test. The results indicated that the missing observations were Missing Completely at Random (MCAR) (*p* > 0.05). Because the proportion of missing data was relatively small and the MCAR assumption was satisfied, no imputation procedures were applied. Instead, listwise deletion was performed, resulting in the exclusion of 31 observation days with incomplete records. Under the MCAR assumption, listwise deletion provides unbiased parameter estimates and is considered an appropriate approach for handling missing data. The final analysis included 761 complete observation days. The excluded observation days were distributed throughout the study period and were not concentrated during specific seasons, public holidays, or unusually high-demand periods. Therefore, listwise deletion was unlikely to introduce meaningful seasonal or temporal bias into the final analytical dataset.

### 3.2. Data Preprocessing

The Classification and Regression Tree (CRT) algorithm in SPSS 29.0 is used to evaluate the nursing workload in the emergency infusion room and analyze the predictor importance and classification stratification rules. The dependent variable was daily chair utilization rate, which served as a proxy measure of nursing workload intensity in the emergency infusion room. This indicator was selected because it reflects the combined influence of patient volume, infusion duration, patient turnover, and operational demand on available nursing resources. The remaining workload metrics collected are used as independent variables.

The CRT analysis automatically identifies hierarchical relationships between variables, generating an optimized decision tree based on predictor significance.

For the regression tree predicting continuous nursing workload, the splitting criterion was based on variance reduction (also termed impurity reduction for regression), which measures the decrease in within-node variance achieved by partitioning a parent node into two child nodes.

Gain Calculation Formula: For a given split *s* at node *t*, the gain $∆\left(s,t\right)$ is computed as:

$$∆\left(s,t\right)=SS\left(t\right)-\left[SS\left(t_{L}\right)+SS\left(t_{R}\right)\right]$$

where:

- $SS\left(t\right)=\sum_{i\in t}{\left(y_{i}-{\bar{y}}_{t}\right)}^{2}$ is the sum of squared deviations from the mean in the parent node;
- $SS\left(t_{L}\right)$ and $SS\left(t_{R}\right)$ are the sums of squared deviations in the left and right child nodes, respectively.

The gain percentage reported for each node represents the proportion of the total variance explained by that split, normalized across all splits in the tree. Specifically, it is calculated as:

$$Gain\%=\frac{∆\left(s,t\right)}{SS_{total}}\times 100\%$$

where $SS_{total}$ is the total sum of squares of the dependent variable.

To avoid potential misunderstanding, it should be noted that negative node means observed in the raw CRT output do not represent negative nursing workload or negative nursing time. These values arise from the internal scaling and calculation procedures used during model construction and reflect relative deviations from the overall sample mean rather than actual workload levels. For presentation purposes, all workload values reported in this study were converted back to their original scale, ensuring that the results can be interpreted directly in terms of workload intensity.

### 3.3. Development of the Decision Tree Model

The CRT algorithm can be used for classification and regression. The output of a classification tree is the category of the sample, while that of a regression tree is a real number. It comprises three steps: feature selection, recursive construction of decision tree, and decision tree pruning.

The CRT model was implemented using binary recursive partitioning in SPSS. Surrogate splitting was not enabled because missing data were addressed prior to model development. Tree complexity was controlled through pre-pruning parameters, including a maximum depth of three levels, a minimum parent node size of 100 observations, and a minimum child node size of 50 observations. Cost-complexity post-pruning was not applied.

Maximum depth = 3: During cross-validation, tree depths ranging from 2 to 10 were evaluated. Depth 3 yielded the optimal trade-off between predictive performance (minimizing cross-validated MSE) and interpretability. Depths greater than 3 produced marginal improvements in accuracy (≤2%) but generated substantially more complex rules, reducing clinical usability. Depth 2, while simpler, resulted in a 12% increase in prediction error. Thus, depth 3 was selected as the parsimonious optimum.

Minimum parent node size =100 and minimum child node size = 50: These parameters control the minimum number of cases required for a node to be split (parent) and for a terminal node (child). These values were optimized via grid search during cross-validation. The selected thresholds ensure that each split is based on a sufficiently large sample (≥100 shifts) to yield stable, generalizable patterns, while preventing splits that create tiny, potentially overfitted terminal nodes (minimum 50 shifts). These sizes represent approximately 15% and 7.5% of the training sample, respectively, striking a balance between capturing meaningful subgroups and avoiding noise-driven splits.

### 3.4. Comparative Model Analysis

To benchmark predictive performance, multiple linear regression, random forest, and gradient boosting models were developed using the same predictor set and evaluated using 10-fold cross-validation. Model performance was compared using R^2^, RMSE, MAE, and MSE.

## 4. Results

### 4.1. Descriptive Statistics of the Independent Variable

Table A1 shows the final inclusion of 761 days of infusion suite workload data, containing 10 representative workload indicators, along with descriptive data.

#### Output of the Decision Tree Model

**Figure 1:**
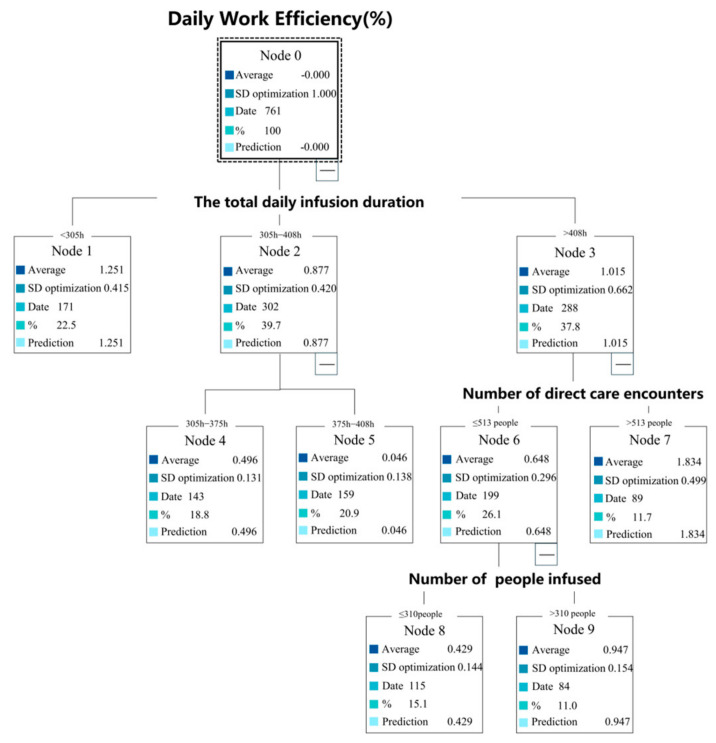
Decision Tree for Infusion Suite Workload Indicators Tree Chart.

The CRT algorithm generated a parsimonious tree with a maximum depth of three, yielding six terminal nodes that classify shift-level workload into low, medium, and high categories. Figure 1 presents the visual structure of the decision tree, with splitting rules and threshold values at each node.

Root Node Split: The total daily infusion duration emerged as the most important predictor, forming the initial partition of the data. Shifts with total infusion duration ≤ 305 h were classified directly into the low workload stratum, representing periods with minimal nursing time requirements.

Secondary Splits for Intermediate Infusion Durations: For shifts with infusion duration between 305 and 408 h, workload stratification required additional predictors. At this node, number of direct care encounters became the decisive factor: shifts exceeding 513 direct care encounters were classified as high workload, while those below this threshold proceeded to the next level.

Tertiary Split for Patient Volume: Among shifts with infusion duration in the intermediate range (305–408 h) and direct care encounters ≤ 513, number of patients receiving infusions provided the final differentiation. Shifts with patient volume > 310 were classified as high workload, whereas those with ≤310 patients were categorized as medium workload.

High-Duration Direct Classification Stratification: Shifts with total infusion duration > 408 h were classified directly as high workload without further splitting, indicating that extreme infusion duration alone is sufficient to predict peak nursing demand.

Figure 1 presents the CRT model illustrating the hierarchical structure of the decision tree.

Table 1 presents the gain statistics for each terminal node of the CRT model. In CRT analysis, the gain percentage reflects the proportion of the overall target outcome captured by a specific node relative to the entire dataset. Higher gain percentages indicate that a node contains a greater concentration of observations associated with higher workload intensity and therefore contributes more substantially to distinguishing workload patterns within the model.

Among the terminal nodes, Node 1 exhibited the highest gain percentage (22.5%) with a mean workload-intensity value of 1.251, indicating that this subgroup accounted for a relatively large share of the overall workload burden identified by the model. In contrast, Node 9 showed the lowest gain percentage (11.0%) with a mean value of 0.947, suggesting a comparatively smaller contribution to the overall workload distribution.

Table 2 presents risk evaluation metrics derived from a decision tree model, comparing two validation methods: *resubstituting* and *10-fold cross-validation*. The resubstituting error (0.045) represents the in-sample risk of the model, indicating how well the decision tree fits the training data.

The close agreement between the re-substitution estimate (0.045, SE = 0.005) and cross-validation estimate (0.046, SE = 0.005) suggests stable predictive performance, supports the model’s generalizability, and indicates limited evidence of overfitting.

To provide a more comprehensive assessment of model performance, additional regression metrics were calculated for the CRT model. The model achieved an R^2^ of 0.939, indicating that approximately 93.9% of the variance in workload intensity was explained by the predictors included in the model. The corresponding MAE, MSE, and RMSE values were 0.035, 0.045, and 0.212, respectively, suggesting good predictive performance on the training dataset (Table 3).

### 4.2. Importance of Variables

**Figure 2:**
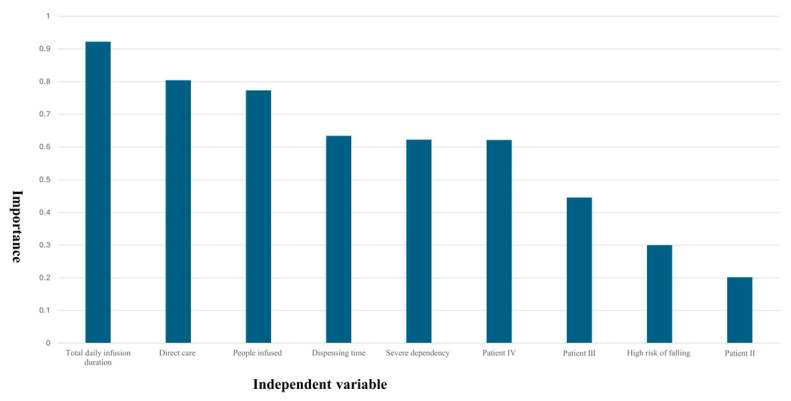
Importance of Independent variable.

Figure 2 presents the relative importance of independent variables in the decision tree model, ranked by their contribution to the outcome prediction. The importance values are expressed as percentages, reflecting the impact of each variable on the decision-making process of the model. The highest importance score (0.922) indicates that total daily infusion duration was the most influential predictor within the fitted CRT model. Direct care encounters (0.804) and the number of patients receiving infusions (0.773) followed closely, suggesting that these variables contributed substantially to workload-intensity prediction within the model.

Medication dispensing time (0.634), severe dependency (0.622), and patient IV volume (0.621) demonstrated moderate predictive importance. Their similar importance scores suggest comparable contributions to the decision-tree splitting process.

Patient III (0.446), high risk of falling (0.300), and patient II (0.202) have a lesser effect. For example, the high risk of falling (32.5%) may affect classification stratification only in specific branches.

### 4.3. Comparative Model Analysis

To benchmark the predictive performance of the CRT model, additional models including multiple linear regression, random forest, and gradient boosting were developed using the same predictor set and evaluated using 10-fold cross-validation. As shown in Table 4, alternative models achieved higher predictive accuracy than the CRT model. However, the CRT model was retained because it provides transparent threshold-based decision rules that can be readily translated into staffing and workload management strategies in emergency infusion rooms.

### 4.4. Sensitivity Analysis

To assess the potential influence of feature leakage between daily chair utilization rate and total infusion duration, a sensitivity analysis was conducted by excluding total infusion duration from the predictor set and rebuilding the CRT model using the same modeling parameters. As shown in Table 5, the sensitivity model demonstrated a reduction in predictive performance compared with the original model. The cross-validated R^2^ decreased from 0.927 to 0.850, and the RMSE decreased from 0.212 to 0.073. Despite this decline in accuracy, the model retained acceptable predictive performance. Direct care encounters remained the most influential predictor, followed by the number of patients receiving infusions and medication dispensing time.

Furthermore, the overall hierarchical structure of the decision tree remained largely consistent, indicating that the workload stratification patterns identified by the CRT model were not solely dependent on total infusion duration. These findings suggest that although total infusion duration contributed substantially to model performance, the main conclusions of the study remained robust after its exclusion.

## 5. Discussion

### 5.1. Interpretation of Key Predictors

The CRT model identified total infusion duration, direct care encounters, and the number of patients receiving infusions as the most influential predictors of workload intensity within the fitted model. These findings are clinically plausible because each indicator captures a distinct dimension of operational demand in emergency infusion rooms.

Total infusion duration reflects cumulative chair occupancy and sustained nursing supervision requirements. Longer infusion times increase the duration of patient monitoring, medication management, and clinical observation, thereby contributing to higher workload intensity. Direct care encounters represent the frequency of nurse–patient interactions and are closely associated with nursing labor input. Similarly, the number of patients receiving infusions reflects overall service volume and operational demand. Together, these predictors capture temporal, activity-based, and volume-related dimensions of workload generation.

The present findings are broadly consistent with previous nursing-workload studies reporting that patient volume, care intensity, and nursing activity levels are major contributors to workload demand. For example, Ivziku et al. [10] identified patient acuity, staffing requirements, and clinical activities as important workload predictors, while Griffiths et al. reported associations between workload variation and patient-care outcomes. Consistent with these studies, total infusion duration, direct care encounters, and patient volume emerged as the most influential predictors within the present model. However, unlike conventional regression-based approaches that primarily estimate associations, the CRT model identified operational thresholds that stratified workload intensity into distinct workload strata, providing a more interpretable framework for management decision-making.

The identified thresholds provide operationally meaningful workload strata that may facilitate workload monitoring and staffing decisions.

### 5.2. Implications for Nursing Management

One important advantage of the CRT model is its ability to generate transparent workload stratification rules [24]. Unlike conventional regression models that primarily quantify statistical associations, the decision tree identifies operational thresholds that can be readily interpreted and translated into management actions.

For example, the CRT model identified specific thresholds for total infusion duration and direct care encounters that distinguished low-, moderate-, and high-workload strata. Because these indicators are routinely collected during daily operations, they could potentially be incorporated into workload-monitoring systems without requiring additional data collection. When workload indicators approach or exceed predefined thresholds, nurse managers may anticipate periods of increased workload demand and consider temporary staffing adjustments, task redistribution, or workforce reinforcement. Conversely, during lower-demand periods, staffing resources may be allocated more efficiently while maintaining patient-care quality.

In addition, the workload stratification framework may provide an objective reference for interpreting differences in workload exposure when evaluating staff performance and resource utilization. Rather than relying solely on patient volume or staffing ratios, managers may use workload strata to contextualize operational demands across different shifts or observation periods.

It should be noted that the present study did not directly evaluate patient outcomes, nursing burnout, staffing efficiency, or the effectiveness of workload-informed staffing interventions. Therefore, the potential management benefits described above should be interpreted as practical applications of the proposed framework rather than outcomes demonstrated by the current study. Future prospective studies are needed to evaluate whether integrating workload stratification into routine management processes improves staffing decisions and operational performance.

### 5.3. Comparison with Previous Studies and Alternative Models

The findings of this study are consistent with previous research indicating that workload intensity is influenced by patient volume, care complexity, and nursing activity levels. Similar predictors have been identified in studies examining emergency department operations, nursing workload measurement, and healthcare resource utilization [33].

To further evaluate model performance, multiple linear regression, random forest, and gradient boosting models were developed using the same predictor set. Although these alternative models achieved higher predictive accuracy than the CRT model, the observed differences in prediction error were relatively modest and did not substantially alter the overall workload stratification patterns identified in this study. From a nursing management perspective, the primary objective of the proposed framework is to support workload categorization and staffing allocation rather than to generate highly precise numerical predictions. Therefore, the improved interpretability and explicit threshold-based decision rules provided by the CRT model may outweigh the modest reduction in predictive accuracy for routine operational decision-making. Nevertheless, ensemble-learning approaches combined with explainable artificial intelligence techniques, such as SHAP or LIME, may further improve predictive performance while preserving interpretability and should be explored in future research.

### 5.4. Strengths and Limitations

This study has several strengths. The use of an interpretable machine-learning approach enabled the identification of workload-related patterns within a real-world emergency infusion setting. In addition, the CRT model generated transparent workload stratification rules that may facilitate operational decision-making and staffing management. The robustness of the findings was further evaluated through comparative analyses and sensitivity analyses.

Several limitations should also be acknowledged. The study was conducted in a single tertiary hospital, which may limit the generalizability of the findings to other healthcare settings. Furthermore, daily chair utilization rate was used as a proxy measure of workload intensity rather than a direct measure of nursing workload. Although this indicator reflects multiple dimensions of operational demand, it may not fully capture all aspects of nursing activities.

A further limitation is that the CRT model was developed using a predefined set of routinely collected operational indicators. Although these variables captured important dimensions of workload demand, additional factors that may influence nursing workload, such as staffing composition, nurse experience, patient-acuity characteristics, workflow interruptions, and organizational factors, were not available for analysis. Consequently, the identified workload stratification rules should be interpreted within the context of the variables examined in this study.

In addition, only internal validation was performed in the present study. Finally, this was a retrospective single-center study conducted in one tertiary emergency infusion room. Therefore, the identified workload predictors, decision thresholds, and workload stratification rules should be interpreted within the context of the present study setting. External validation using multicenter datasets and alternative workload measures is warranted before broader clinical implementation can be recommended.

## 6. Conclusions

This study developed and evaluated an interpretable classification and regression tree (CRT) model to assess workload intensity in an emergency infusion room using routinely collected operational indicators. Within the fitted model, total infusion duration, direct care encounters, and the number of patients receiving infusions emerged as the most influential predictors of workload intensity, represented by daily chair utilization rate.

The CRT model identified transparent threshold-based workload stratification patterns and demonstrated acceptable predictive performance. Comparative analyses showed that alternative models, including multiple linear regression, random forest, and gradient boosting, achieved higher predictive accuracy. However, the CRT model provided greater interpretability and generated explicit decision rules that may be more readily translated into nursing management practice.

Sensitivity analyses further suggested that the overall workload stratification pattern remained relatively stable after excluding total infusion duration, supporting the robustness of the main findings. Nevertheless, the use of daily chair utilization rate as a proxy measure of workload intensity and the potential conceptual overlap with infusion duration should be considered when interpreting the results.

Overall, the proposed framework provides a practical and interpretable approach for workload assessment in emergency infusion settings and may support data-informed staffing allocation and operational decision-making. Future studies should validate the model across multiple institutions and explore alternative workload outcome measures to further enhance generalizability and applicability.

## Figures and Tables

**Table 1:** Summary of node gains.

Node	*n*	%	Mean Value
7	89	11.7%	1.834
9	84	11.0%	0.947
8	115	15.1%	0.429
5	159	20.9%	0.046
4	143	18.8%	0.496
1	171	22.5%	1.251

**Table 2:** Risk for the decision tree.

Method	Estimation	Standard Error
Re-substitute	0.045	0.005
Cross-validation	0.046	0.005

**Table 3:** Performance metrics of the CRT model.

Metric	Training Set
R^2^	0.939
MAE	0.035
MSE	0.045
RMSE	0.212

**Table 4:** Comparative predictive performance of different modeling approaches based on 10-fold cross-validation.

Model	R^2^	RMSE	MAE
Linear Regression	1.000	<0.001	<0.001
Random Forest	0.993	0.015	0.008
Gradient Boosting	0.999	0.007	0.005
CRT	0.927	0.212	0.037

**Table 5:** Sensitivity analysis excluding total infusion duration.

Model	R^2^	MSE	RMSE	MAE
Original CRT model	0.927	0.045	0.212	0.037
Excluding total infusion duration	0.850	0.039	0.073	0.053

## Data Availability

The datasets used and/or analyzed during the current study are available from the corresponding author on reasonable request.

## Appendix A

**Table A1:** Descriptive statistics.

	N	Scope	Minimum Value	Maximum Value	Value		Standard Deviation	Variance	Skewness		Kurtosis	
Independent Variable	Statistic	Statistic	Statistic	Statistic	Statistic	Standard Error	Statistic	Statistic	Statistic	Standard Error	Statistic	Standard Error
Total daily infusion duration	761	35.85	1.10	36.95	17.3163	0.23258	6.41611	41.166	0.365	0.089	0.197	0.177
Dispensing time	761	6.87	0.03	6.90	1.5623	1.10570	1.10570	1.223	1.749	0.089	3.435	0.177
People infused	761	354	13	367	183.98	59.542	59.542	3545.290	0.118	0.089	0.168	0.177
Direct care	761	1055	39	1094	559.99	184.904	184.904	34,189.531	0.138	0.089	0.226	0.177
Patient II	761	15	0	15	4.01	2.917	2.917	8.507	1.157	0.089	1.447	0.177
Patient III	761	98	2	100	35.79	18.699	18.699	349.665	0.703	0.089	0.142	0.177
Patient IV	761	294	11	305	144.12	46.429	46.429	2155.688	0.342	0.089	0.175	0.177
High risk of falling	761	40	0	40	10.51	6.557	6.557	43.000	1.209	0.089	1.626	0.177
Severe dependency	761	196	5	200	75.68	34.200	34.200	1169.612	0.536	0.089	0.133	0.177
Effective cases	761											

## References

[1] Swiger P.A., Vance D.E., Patrician P.A. (2016). Nursing workload in the acute-care setting: A concept analysis of nursing workload. Nurs. Outlook.

[2] Hellín Gil M.F., Roldán Valcárcel M.D., Seva Llor A.M., Ibáñez-López F.J., Mikla M., Montesinos M.J.L. (2022). Validation of a nursing workload measurement scale based on the classification of nursing interventions for adult hospitalization units. Int. J. Environ. Res. Public Health.

[3] De Groot K., De Veer A.J.E., Munster A.M., Francke A.L., Paans W. (2022). Nursing documentation and its relationship with perceived nursing workload: A mixed-methods study among community nurses. BMC Nurs..

[4] Hellín Gil M.F., Mikla M., Seva Llor A.M., Valcárcel M.D.R., Ibáñez-López F.J., Montesinos M.J.L. (2022). Multicenter application of a nursing workload measurement scale in adult hospitalization units. Int. J. Nurs. Sci..

[5] Mohammadnejad F., Freeman S., Klassen-Ross T., Hemingway D., Banner D. (2023). Impacts of technology use on the workload of registered nurses: A scoping review. SAGE Open Nurs..

[6] Sánchez-Sánchez M.M., Campos-Asensio C., Arias-Rivera S. (2024). Workloads of intensive care nurses: Validity of their estimation using mobile applications and comparison with Nursing Activities Score. Enfermería Intensiv..

[7] Home J.H.C.A., Zhao J., Sherman B., McCracken A., Watkins S., Rusinko K. (2023). Development of Productivity Standards for Ambulatory Infusion Suite Nurses Within a Multi-Entity Health System. Infus. J..

[8] Milo A. (2020). How Much Room Is Left? Understanding Infusion Capacity by Defining Utilization. Oncol. Pract. Manag..

[9] LeanTaaS (2017). Scheduling Tool Improves Infusion Center Efficiency. https://www.targetedonc.com/view/scheduling-tool-improves-infusion-center-efficiency.

[10] Ivziku D., Clari M., Piredda M., Matarese M., Messina M.P., Albanesi B., De Marinis M.G. (2022). Defining Nursing Workload Predictors: A Pilot Study. J. Nurs. Manag..

[11] Maghsoud F., Sadeghi M., Hosseinzadeh M., Peyrovi H. (2022). The Mediating Role of Implicit Rationing of Nursing Care, Job Satisfaction, and Emotional Exhaustion in the Relationship between Workload and Quality of Nursing Care. BMC Nurs..

[12] Sendak M.P., D’Arcy J., Kashyap S., Gao M., Nichols M., Corey K., Ratliff W., Balu S. (2020). A Path for Translation of Machine Learning Products into Healthcare Delivery. EMJ Innov..

[13] Mahmoudi E., Kamdar N., Kim N., Gonzales G., Singh K., Waljee A.K. (2020). Use of Machine Learning in Predicting Healthcare Utilization. Health Serv. Res..

[14] Kang J., Kwon S.S., Lee Y. (2024). Clinical nurses’ work-life balance prediction due to patient safety incidents using classification and regression tree analysis: A secondary data analysis. BMC Nurs..

[15] Song Y., Zhang X., Luo D., Shi J., Zang Q., Wang Y., Yin H., Xu G., Bai Y. (2024). Predicting nursing workload in digestive wards based on machine learning: A prospective study. BMC Nurs..

[16] El Ariss A.B., Kijpaisalratana N., Ahmed S., Yuan J., Coleska A., Marshall A., Luo A.D., He S. (2024). Development and validation of a machine learning framework for improved resource allocation in the emergency department. Am. J. Emerg. Med..

[17] Moreno-Sánchez P.A., Aalto M., van Gils M. (2024). Prediction of patient flow in the emergency department using explainable artificial intelligence. Digit. Health.

[18] Hu Y., Chan C.W., Dong J., Kazekjian A., Ophaswongse C., Sugalski G., Underwood J.P., Perotte R. (2025). Implementing a prediction driven framework for emergency department nurse staffing to optimize real time decisions. npj Health Syst..

[19] Arrieta A.B., Díaz-Rodríguez N., Del Ser J., Bennetot A., Tabik S., Barbado A., García S., Gil-López S., Molina D., Benjamins R. (2020). Explainable Artificial Intelligence (XAI): Concepts, taxonomies, opportunities and challenges toward responsible AI. Infin. Fusion.

[20] Amann J., Blasimme A., Vayena E., Frey D., Madai V.I. (2020). Explainability for artificial intel-ligence in healthcare: A multidisciplinary perspective. BMC Med. Inform. Decis. Mak..

[21] Tjoa E., Guan C. (2021). A survey on explainable artificial intelligence (XAI): Toward medical XAI. IEEE Trans. Neural Netw. Learn. Syst..

[22] Porto B.M. (2024). Improving triage performance in emergency departments using machine learning and natural language processing: A systematic review. BMC Emerg. Med..

[23] Tohka J., van Gils M. (2021). Evaluation of machine learning algorithms for health and wellness applications: A tutorial. Comput. Biol. Med..

[24] Griffiths P., Saville C., Ball J.E., Jones J., Pattison N., Monks T. (2020). Nursing workload, nurse staffing methodologies and tools: A systematic scoping review and discussion. Int. J. Nurs. Stud..

[25] Lin H., Wen Y., Liu T., Liao S., Ma W. (2017). Application of performance appraisal in infusion room using PDA as a platform. China Med. Her..

[26] Su J. (2017). Application effect of quantitative assessment in nursing management of infusion room in emergency department. J. Chin. Med. Manag..

[27] Gao J., Cao Y., He B., Hao W. (2021). Construction and application of MNIS combined with weight coefficients as a nursing workload measurement system in infusion room. China Clin. Res..

[28] Mafirakureva N., Denoeud-Ndam L., Tchounga B.K., Otieno-Masaba R., Herrera N., Mukherjee S., Casenghi M., Tiam A., Dodd P.J., INPUT Study Group (2024). Cost-effectiveness of integrating paediatric tuberculosis services into child healthcare services in Africa: A modelling analysis of a cluster-randomised trial. BMJ Glob. Health.

[29] Tesfa G.A., Demeke A.D., Seboka B.T., Tebeje T.M., Kasaye M.D., Gebremeskele B.T., Hailegebreal S., Ngusie H.S. (2024). Employing machine learning models to predict pregnancy termination among adolescent and young women aged 15–24 years in East Africa. Sci. Rep..

[30] Chi J.-T., Song Z.-L., Zhu Y.-J., Xie J.-M., Wang X.-N., Zhou L. (2019). Application of decision tree classification method in nursing workload evaluation in surgical wards. Chin. J. Pract. Nurs..

[31] Topol E.J. (2019). High-performance medicine: The convergence of human and artificial intelligence. Nat. Med..

[32] Lundberg S.M., Erion G., Chen H., DeGrave A., Prutkin J.M., Nair B., Katz R., Himmelfarb J., Bansal N., Lee S.I. (2020). From local explanations to global understanding with explainable AI for trees. Nat. Mach. Intell..

[33] Morris R., MacNeela P., Scott A., Treacy P., Hyde A. (2021). Reconsidering the conceptualization of nursing workload: A literature review. J. Adv. Nurs..

